# Ordered Mesoporous Silica Prepared in Different Solvent Conditions: Application for Cu(II) and Pb(II) Adsorption

**DOI:** 10.3390/gels8070443

**Published:** 2022-07-15

**Authors:** Ana-Maria Putz, Oleksandr I. Ivankov, Alexander I. Kuklin, Vasyl Ryukhtin, Cătălin Ianăşi, Mihaela Ciopec, Adina Negrea, László Trif, Zsolt Endre Horváth, László Almásy

**Affiliations:** 1 “Coriolan Drăgulescu” Institute of Chemistry, Bv. Mihai Viteazul, No. 24, 300223 Timisoara, Romania; putzanamaria@acad-icht.tm.edu.ro (A.-M.P.); cianasic@yahoo.com (C.I.); 2 Frank Laboratory of Neutron Physics, Joint Institute for Nuclear Research, Joliot-Curie 6, 141980 Dubna, Russia; ivankov@jinr.ru (O.I.I.); kuklin@jinr.ru (A.I.K.); 3 Nuclear Physics Institute, ASCR, Husinec—Řež 130, 250 68 Řež, Czech Republic; ryukhtin@ujf.cas.cz; 4 Faculty of Industrial Chemistry and Environmental Engineering, Politehnica University Timişoara, 6th Vasile Pârvan Bvd., 300223 Timisoara, Romania; mihaela.ciopec@upt.ro (M.C.); adina.negrea@chim.upt.ro (A.N.); 5 Institute of Materials and Environmental Chemistry, Research Centre for Natural Sciences, Magyar Tudósok Körútja 2, 1117 Budapest, Hungary; trif.laszlo@ttk.hu; 6 Institute for Technical Physics and Material Science, Centre for Energy Research, Konkoly-Thege Miklós út 29-33, 1121 Budapest, Hungary; horvath.zsolt.endre@ek-cer.hu; 7 Institute for Energy Security and Environmental Safety, Centre for Energy Research, Konkoly-Thege Miklós út 29-33, 1121 Budapest, Hungary

**Keywords:** MCM-41, SANS, USANS, SAXS, Stöber method, 2-methoxyethanol, Langmuir isotherm

## Abstract

In this work, the synthesis of ordered mesoporous silica of MCM-41 type was investigated aimed at improving its morphology by varying the synthesis conditions in a one-pot process, employing different temperatures and solvent conditions. 2-methoxyethanol was used as co-solvent to ethanol. The co-solvent ratio and the synthesis temperature were varied. The pore morphology of the materials was characterized by nitrogen porosimetry and small angle neutron scattering (SANS), and the particle morphology by transmission electron microscopy (TEM) and ultra-small angle neutron scattering (USANS). The thermal behavior was investigated by simultaneous thermogravimetry-differential scanning calorimetry (TG-DSC) measurements. The SANS and N_2_ sorption results demonstrated that a well-ordered mesoporous structure was obtained at all conditions in the synthesis at room temperature. Addition of methoxyethanol led to an increase of the pore wall thickness. Simultaneously, an increase of methoxyethanol content led to lowering of the mean particle size from 300 to 230 nm, according to the ultra-small angle scattering data. The ordered porosity and high specific surfaces make these materials suitable for applications such as adsorbents in environmental remediation. Batch adsorption measurements of metal ion removal from aqueous solutions of Cu(II) and Pb(II) showed that the materials exhibit dominantly monolayer surface adsorption characteristics. The adsorption capacities were 9.7 mg/g for Cu(II) and 18.8 mg/g for Pb(II) at pH 5, making these materials competitive in performance to various composite materials.

## 1. Introduction

Numerous research has dealt with the problem of obtaining suitable adsorbents in order to remove the hazardous pollutants. Adsorption technology is the most promising and frequently used approach due to its simplicity, high efficiency and low cost [1]. Various kinds of sorbent materials, both natural and synthetic, have been used to remove heavy metal ions from aqueous solution. Low cost industrial and agricultural by-products and waste materials usually have low adsorption efficiency because of their low pore volumes and poor pore structure [2,3]. Novel nanomaterials, including carbon-based nanomaterials, metal-oxide based nanomaterials, nanoparticles and various nanocomposites have been systematically applied for the removal of heavy metal ions from wastewater [4,5,6,7,8,9,10,11].

Due to their simplicity and abundance, silica and silica-based sorbents obtained from natural bio or waste sources represent an emerging approach in applications for removal of industrial pollution [12,13,14,15,16,17].

Synthetic silica based mesoporous materials, in spite of their higher costs, have also received wide attention and have been widely used for the adsorption of heavy metal ions due to their exceptionally large specific surface area, regular pore structure and suitability for surface modifications [18,19,20,21].

Silica materials with ordered porosity and open pore network, such as MCM-41 and SBA-15, are especially suitable for capturing species from aqueous solutions. In the classical synthesis of MCM-41-type materials, the silica precursors and the surfactant molecules used for pore templates are dissolved in water-ethanol mixtures. Full or partial replacement of ethanol with another co-solvent can modify the reaction speed of the sol-gel process, and change the morphology of the resulting silica gel [22,23]. The more hydrophobic 2-methoxyethanol has been used previously as a co-solvent in sol-gel synthesis of uniform spherical silica nanoparticles [24]. Hydrophobic organic co-solvents can affect the pore size by mingling with the surfactant molecules and acting as spacers inside the micelles [25,26]. For example, it was proven that ethoxyethanol acts not only as solvent but also as co-surfactant, controlling the morphology and pore structure. In alkali-catalyzed reactions, the co-surfactants promote the water solubility of the silica precursor tetraethoxysilane (TEOS), facilitating the hydrolysis reactions [27,28]. In previous works, 2-methoxyethanol was used as a protic polar solvent playing different specific roles: as solvent and complexing agent in the synthesis of mixed silica–titania [29], or as a solvent as well as a stabilizer of alkoxide in the hydrolysis-precipitation reaction, allowing one to control the reactivity of the precursors by adjustment of the quantity of 2-methoxyethanol [30].

In the present study, we applied a simple sol-gel synthesis procedure to obtain sorbent materials suitable, among other applications, for water pollutant removal. Two series of ordered mesoporous silica were synthetized, one at room temperature and the other one at 50 °C, and different amounts of 2-methoxyethanol were used in the range of 0:1 to 3:1 relative to the ethanol content. Cetyltrimethylammonium bromide (CTAB) was used as a pore forming agent. The influence of the mixed solvent composition and synthesis temperature on the particle morphology and the pore network was studied by small angle neutron scattering and nitrogen porosimetry. 

The applicability of the obtained materials as adsorbents for heavy metals ions from water was studied with the example of Pb(II) and Cu(II).

## 2. Results and Discussion

### 2.1. FT-IR Analysis

**Figure 1:**
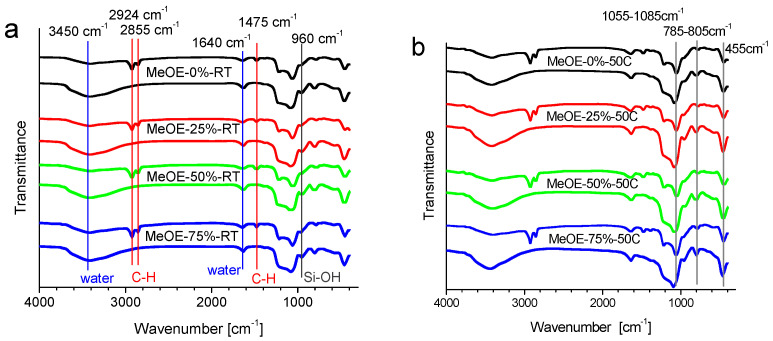
FT-IR spectra of mesoporous silica synthesized at room temperature (**a**) and at 50 °C (**b**), before and after calcination. Vertical lines in panel (**a**) show the characteristic bands of CTAB that disappear after thermal treatment (red); the vibrations related to water (blue) and Si-OH vibrations (dark grey). The vertical lines in panel (**b**) show the characteristic bands of the silica network.

All samples (xerogels and thermally treated materials) show the specific vibration bands for the silica skeleton at 1055–1085 cm^−1^, 785–805 cm^−1^ and 450–455 cm^−1^ (Figure 1). This is assigned to the asymmetric stretching [31,32], symmetric stretching [33,34] and bending rocking mode vibration of the Si-O-Si network [32], respectively. For all calcined samples, the asymmetric stretching vibration bands are shifted to higher values by approximately 20–30 cm^−1^. This effect is related to the densification of the silica network [34]. The 1640 cm^−1^ vibration band belongs to molecular water [35]. The presence of the silanol groups was confirmed by the presence of the band centered about 960 cm^−1^, which is associated with the stretching mode of the Si-OH groups [35]. The broad band centered at around 3440–3405 cm^−1^ corresponds to the O-H stretching bands of hydrogen-bonded water molecules (H-O-H···H) and the Si-O-H stretching of surface silanols hydrogen-bonded to water (-SiOH···H_2_O) [33]. They become more intense after calcination due to the increased hygroscopicity of the calcined samples, because the removal of the surfactant molecules allows some water to enter the pores [36].

Another group of characteristic vibrations is due to surfactant molecules. In the non-calcined samples, the bands at 2924 and 2855 cm^−l^ are the asymmetric and symmetric stretching vibrations of the alkyl chain, respectively, and the band around 1475 cm^−1^, is the bending vibration of the C–H groups [35]. These bands completely disappear after calcination for all samples (Figure 1).

### 2.2. Thermal Analysis

**Figure 2:**
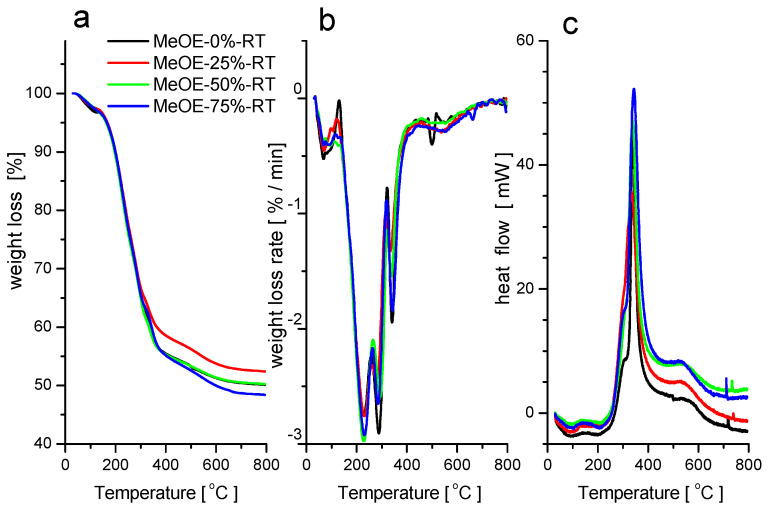
TG (**a**), DTG (**b**) and DSC (**c**) curves for samples prepared at room temperature.

Thermogravimetric and scanning calorimetry analyses in synthetic air were performed on the RT series of xerogels not subjected to calcination. The weight loss curves for the four samples are shown in Figure 2a. On the DTG curves (Figure 2b), five distinct weight loss regions can be identified, from which the second and the third steps are overlapped. In the first temperature range between 25 and 150 °C, physisorbed water evaporates, which is accompanied by a small and broad endotherm (Figure 2c). Between 150 and 350 °C, the surfactant decomposes and burns out. The two overlapped peaks on the DTG curve can be attributed to the separate oxidation and decomposition of the tetramethyl ammonium headgroups and the long alkyl chains of the CTAB. The oxidative burning of the organic matter is accompanied by a sharp and large exothermic signal on the heat flow curves around 340 °C. Between 400 and 700 °C, the small weight loss, accompanied by a small and broad exotherm, can be attributed to the complete oxidation and elimination of the decomposed organic compounds from the pores. 

The spike-like exothermic peaks in the DSC curves at around 720–740 °C indicate phase transformations in the silica matrix.

In their thermal behavior, no systematic differences between the four samples were observed, which can be explained by the small differences in the morphology, and indicate that not much of methoxyethanol remained entrapped in the pores after the washing procedure during synthesis. The total weight loss of about 50% in the whole temperature range corresponds to the typical content of CTAB surfactant in the MCM-41 type materials [36].

### 2.3. Nitrogen Porosimetry

**Figure 3:**
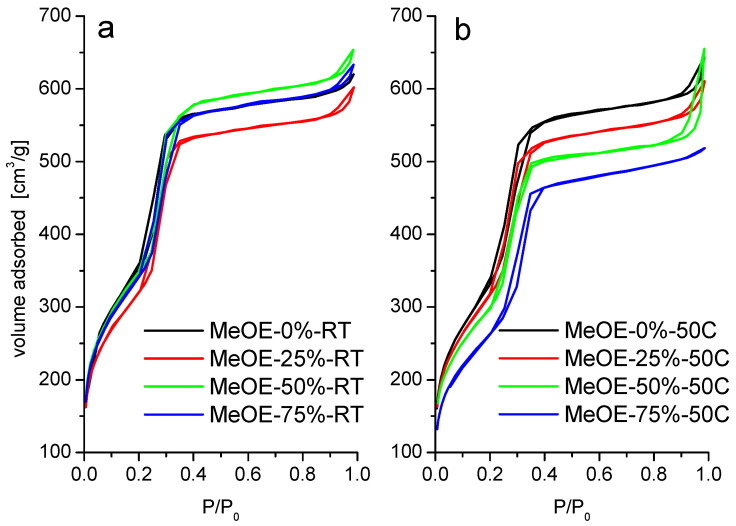
N_2_ adsorption-desorption isotherms of calcined samples prepared at room temperature (**a**) and at 50 °C (**b**).

The nitrogen physisorption isotherms for all materials are shown in Figure 3. The materials present type IVb isotherm according to IUPAC classification. Type IVb is specific for mesoporous materials having conical or cylindrical pores, closed at one end [37,38]. The narrow hysteresis in the interval 0.2–0.4 P/P_0_ suggests the presence of some wide pores that could have more access to the external surface [38].

**Figure 4:**
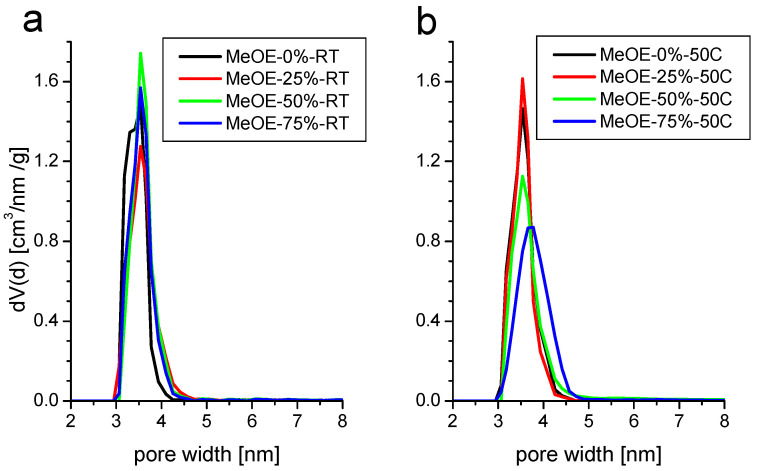
Pore size distribution of samples synthetized at room temperature (**a**) and at 50 °C (**b**).

The pore size distribution (Figure 4) is narrow and centered around 3.5 nm for all samples. The BJH mean pore size shows a small increase from 3.0 to 3.3 nm, with the increase of methoxyethanol concentration in the solvent. The total pore volume does not change appreciably, except for the samples prepared with the highest methoxyethanol content at 50 °C when it drops by about 40%. This is related to the partial collapse of the pores, as shown later by the SANS results. 

The different texture of the materials was also observed in the surface fractal dimensions evaluated by Frenkel-Halsey-Hill (FHH) method [39]. The FHH model is used to determine the fractal geometry and calculate surface structure and irregularities. Value of Df 2 indicates smooth surface and 3 is characteristic for a porous surface [39]. The FHH data (Table 1) indicate that using methoxyethanol as co-solvent a smoother surface is obtained compared to the materials prepared only with ethanol.

Materials synthetized at 50 °C present almost the same characteristics. The isotherms are of type IVb with a narrow hysteresis (Figure 3b). The surface area decreases with increasing methoxyethanol content. For this series, the pore size distribution is somewhat broader, especially for the highest methoxyethanol content (Figure 4b).

All textural parameters for surface area, total pore volume and mean pore diameter are shown in Table 1. The highest BET surface area of 1793 m^2^/g was obtained for sample synthesized at room temperature, and all samples had surface areas above 1000 m^2^/g. With increasing 2-methoxyethanol content in the solvent mixture, the surface area decreased in both series. The changes in the surface area and roughness can be related to the lowering of the speed of hydrolysis and condensation with addition of methoxyethanol having hydrophobic methyl and methylene groups.

### 2.4. Electron Microscopy

**Figure 5:**
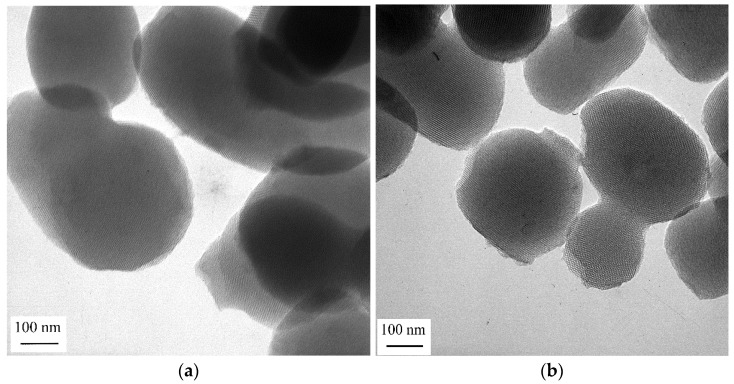
Characteristic TEM images of mesoporous silica prepared at room temperature without 2-methoxyethanol (**a**) and in solvent with 3:1 methoxyethanol/ethanol ratio (**b**).

TEM micrographs of the two investigated samples showed spherical and slightly elongated particles with sizes of 200–400 nm, typical for silica obtained in the Stöber synthesis process (Figure 5). Well-developed parallel channels with hexagonal ordering confirmed the structure of the MCM-41 materials. There were no noticeable differences between the samples MeOE-0%-RT and MeOE-75%-RT, in the selected areas of the TEM images.

### 2.5. Small Angle Neutron Scattering

**Figure 6:**
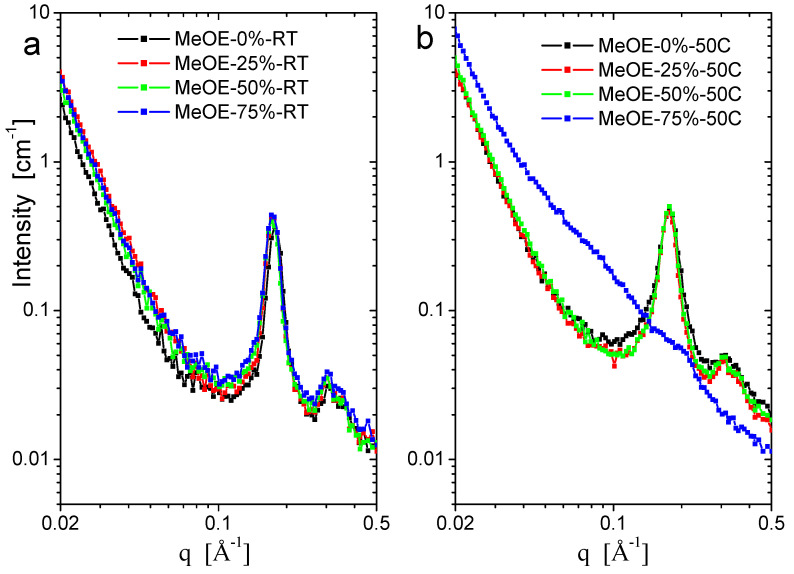
SANS scattering curves of samples prepared at room temperature (**a**) and at 50 °C (**b**).

**Figure 7:**
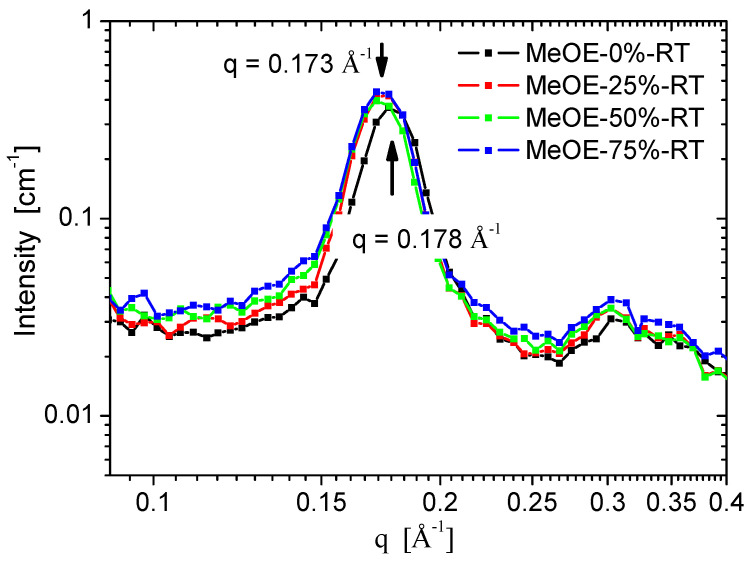
Variation of the first diffraction peak position with the content of 2-methoxyethanol in the solvent mixture.

SANS curves for the two series taken on spectrometer YuMO are shown in Figure 6a,b. All samples show the typical organized structure of MCM-41 type, exhibiting the (100), (110), (200) reflections of the hexagonal pore structure [40,41,42]. The (110) and (200) peaks are smeared because of the moderate instrumental resolution related to the relaxed collimation and finite detector ring size, characteristic for small-angle neutron instruments. The sample prepared at 50 °C using 75% methoxyethanol is disordered (Figure 6b) and the diffraction peaks are nearly invisible. No well-ordered structure formed in the synthesis at these conditions, and the xerogel filled with CTAB was weaker and the pore structure was destroyed during calcination. Another characteristic difference between the samples prepared at the two temperatures is the width of the first diffraction peak, which is broader for the 50 °C synthesis, indicating a weaker long-range ordering. For samples prepared at room temperature, a significant difference was seen in the (100) peak position, between the material prepared in pure ethanol solvent, and the three samples prepared in mixed solvents. The peak position for MeOE-0%-RT sample was smaller by 3%, showing larger interpore distance, which indicates a larger wall thickness (Figure 7). This effect can be related to the slower condensation rate of the silica the presence of 2-methoxyethanol as cosolvent.

**Figure 8:**
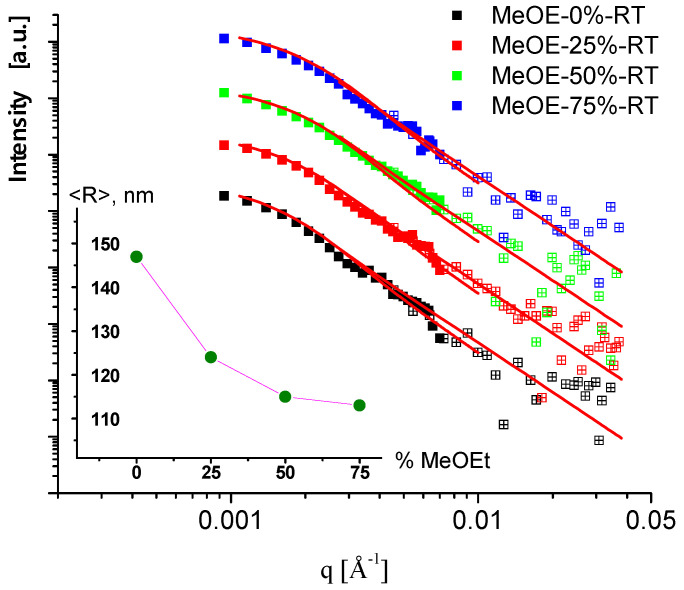
USANS scattering curves of samples prepared at room temperature. Symbols are the measured data, and red solid lines are the fitted model of polydisperse spheres. Data are shifted vertically. In the inset, the mean radius of the particles is shown in the function of 2-methoxyethanol content in the sol.

Measurements of the RT sample series in the very low *q* range, performed on the MAUD instrument, allowed us to assess the overall dimensions of the nanoparticles. The scattering curves measured with the medium and low-resolution instrument settings (solid and open symbols) are shown in Figure 8. The scattering data were fitted to a model of polydisperse spherical particles with log-normal size distribution. The resulting mean diameters were in the range of 200–300 nm, which is typical for Stöber synthesis. Interestingly, a well distinguished monotonous change of the particle size in the function of methoxyethanol content was observed: the larger (300 nm) particles were obtained using pure ethanol-water solvent mixture, and the gradual replacement of ethanol by 2-methoxyethanol resulted in smaller (230 nm) particles.

### 2.6. Metal Ion Adsorption 

Adsorption isotherms are powerful tools for the analysis of adsorption processes. They establish the relationship between the equilibrium concentration and the amount adsorbed by unit mass of the adsorbent at a constant temperature. Adsorption studies were performed at pH 5 because it was shown previously for these kind of materials that the maximum adsorption capacities for Cu^2+^ and Pb^2+^ were observed at pH 5.0 [19]. First, all eight samples were tested for adsorption of Cu(II) and Pb(II), and then the material with the best adsorption capacity for Cu(II), (sample MeOE-75%-RT), and the material with the best adsorption capacity for Pb(II), (sample MeOE-0%-RT) were selected for construction of the adsorption isotherms. The equilibrium time for adsorption was determined varying the time in the range 15–180 min for a Me(II) ions concentration of 10 mg/L. After 2 h, the adsorption capacity has a constant value. Two hours of contact time was used in constructing the adsorption isotherms.

The adsorption capacity *q* (mg/g), was determined using the following equation:

(1) $$q=(C_{0}-C_{f})\frac{V}{m}$$

where *C*_0_ and *C_f_* are the initial and final concentrations of metallic ions in solution in the beginning and the end of the adsorption test, respectively (mg/L), *V* is the volume of the solution (L) and *m* is the mass of the adsorbent (g).

**Figure 9:**
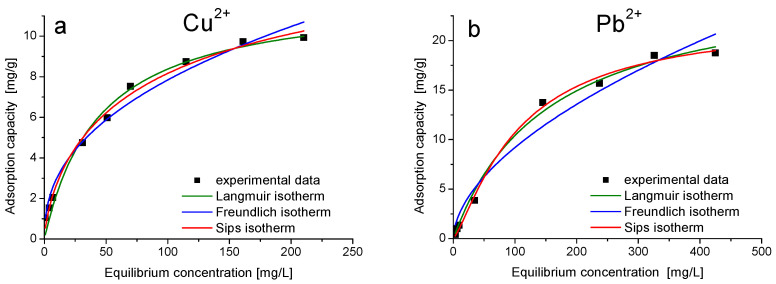
Adsorption isotherms of (**a**) Cu(II) on MeOE-75%-RT and (**b**) Pb(II) on MeOE-0%-RT.

The adsorption isotherms of Me(II) ions are presented in Figure 9. The Langmuir, Freundlich and Sips isotherms were used to model experimental data in order to establish the adsorption mechanism and the maximum adsorption capacity [43,44].

The non-linear form of the Langmuir isotherm equation [45] can be expressed as follows:

(2) $$q_{e}=\frac{q_{L}\;K_{L}\;C_{f}}{1+K_{L}\;C_{f}}$$

where *q_e_* is the equilibrium absorption capacity (mg/g); *C_f_* is the equilibrium ion concentration in solution (mg/L); *q_L_* is the Langmuir maximum adsorption capacity (mg/g) and *K_L_* is the Langmuir constant.

The Freundlich isotherm is applicable to heterogeneous adsorption surfaces. The non-linear form of the Freundlich isotherm equation [46] is:

(3) $$q_{e}=K_{F}\;C_{f}^{1/n_{f}}$$

where *K_F_* and *n_f_* are the characteristic constants related to the adsorption capacity of the adsorbent and the intensity of adsorption.

The Sips or Langmuir-Freundlich isotherm (Equation (4)) derives from the Freundlich and Langmuir models. It reduces to a Freundlich isotherm at low adsorbate concentrations. At high adsorbate concentrations, the Sips isotherm has the characteristics of the Langmuir isotherm and therefore can be used to calculate the monolayer adsorption capacity [47].

(4) $$q_{e}=\frac{q_{S}\;K_{S}\;C_{e}^{1/n_{S}}}{1+K_{S}\;C_{e}^{1/n_{S}}}$$

where *K_S_* (mg/g) is a constant related to the adsorption capacity of the adsorbent and *n_s_* is the heterogeneity factor. As the surface heterogeneity is higher, the deviation of 1/*n_s_* value from 1 will be higher. The parameters of Langmuir, Freundlich and Sips isotherms determined using non-linear regression are listed in Table 2.

The adsorption capacity increased with increasing equilibrium concentration of Me(II) ions. The experimental isotherms show the approach of the adsorption capacity to constant value and, correspondingly, the large deviation of the fitted Freundlich model curves from the data. The maximum adsorption capacity of Cu(II) on sample MeOE-75%-RT for an initial Cu(II) concentration of 250 mg/L is *q*_m_,_exp_ = 9.7 mg/g, and the maximum adsorption capacity of Pb(II) on sample MeOE-0%-RT is *q_m_*,_exp_ = 18.8 mg/g for an initial Pb(II) concentration of 500 mg/L.

For both materials, the adsorption is better described by the Sips isotherm. The maximum adsorption capacity obtained by modelling the experimental data with Sips isotherm is *q*_s_= 10.2 mg Cu(II)/g sorbent, a value very close to the experimental value *q*_m,exp_ = 9.7 mg Cu(II)/g sorbent for sample MeOE-75%-RT and *q*_s_ = 22.2 mg/g, a value very close to the experimental value *q*_m,exp_ = 18.8 mg Pb(II)/g for sample MeOE-0%-RT at the highest salt concentration. Parameter 1/*n* in the Sips model is a measure of the adsorption intensity or surface heterogeneity. For 1/*n* = 1, the partition between the two phases is independent of the concentration. The situation 1/*n* < 1 is the most commonly encountered and corresponds to Langmuir isotherm, while 1/*n* > 1 indicates cooperative adsorption involving strong interactions between the molecules of the adsorbate [48,49]. The value of the heterogeneity factor 1/*n_s_* was obtained as 0.3 for sample MeOE-75%-RT and 0.6 for sample MeOE-0%-RT, indicating that the heterogeneity of the surface was very low. This suggests that the adsorption mechanism approaches the monolayer adsorption and the adsorption of Me(II) ions onto adsorbent materials was a favourable process.

The maximum adsorption capacities of these materials were compared to some other adsorbents from recent literature (Table 3). Concerning the Pb(II) adsorption, the obtained value of 18.8 mg/g proved that our mesoporous silica has better adsorption properties compared with kaolin, activated carbon and zeolite, but worse than the functionalized mesoporous silica and the composite materials of iron oxide nanoparticles with functionalized porous silica or cellulose (Table 3). Concerning the Cu(II) adsorption, the obtained value of 9.7 mg/g for the adsorption capacity is higher than that of zeolites, similar to magnetite nanoparticles, but worse than the adsorption capacities of other metal oxide nanoparticles or mesoporous silica having different functional groups. Therefore, the functionalization of mesoporous silica materials is necessary to improve their adsorption capacity for metal ions removal. On the other hand, the ease of preparation and low cost of the nonfunctionalized mesoporous silica may compensate for their lower performance in certain applications.

## 3. Conclusions

In this paper we studied morphology evolution of ordered mesoporous silica nanoparticles of MCM-41 type, prepared at different solvent conditions using 2-methoxyethanol as a co-solvent and CTAB as a pore forming molecule, by conventional pin-hole and slit-smeared ultra-small-angle neutron scattering. The use of mixed solvent resulted in larger wall thickness and interpore distance, and the hexagonal mesoporous structure was maintained up to 75/25 methoxyethanol/ethanol ratio. This effect can be related to the slower condensation rate of the silica network in the presence of 2-methoxyethanol as cosolvent. These structural changes were accompanied by a 10% decrease of the surface area, as measured by nitrogen sorption. A weaker long-range ordering was obtained for the samples synthesized at elevated temperature of 50 °C. These textural and structural observations show that varying the solvent conditions and temperature allows one to tune and optimize the porosity and intrapore surface of the materials, which is of primary importance for their applications as sorbent materials. In future work the effect of other synthesis conditions should be explored aiming to achieve the highest porosity, high specific surface and surface quality modification by functional groups, for achieving efficient binding of metal and organic pollutants.

Selected samples were tested for adsorption of metal pollutants from aqueous phase. The adsorption equilibrium data of Pb(II) and Cu(II) followed the Sips and the Langmuir isotherms, while the Freundlich model did not fit the experimental data. The modeling showed that the heterogeneity of the adsorbent materials surface was very low, suggesting that the adsorption mechanism approached monolayer adsorption. Comparison of the adsorption performance of the prepared materials to various adsorbents from the literature revealed that the adsorption capacity of the present mesoporous silica is comparable to some simple materials, but lower than many of the much more expensive composite materials.

This study showed that while the modification of the structure, overall dimension and pore size can be tuned easily within narrow limits by changing the solvent composition, the resulting effect on the adsorption performance is not particularly strong within the investigated synthesis conditions. This is attributed to the relatively small change of the pore size and internal surface by the simple solvent variation method. Replacement of the traditional CTAB with longer or shorter alkyl chain surfactants can efficiently vary the porosity within a certain range. Furthermore, using more eco-friendly biosurfactants may be beneficial in long term, after resolving the most prohibitive factors such as their high costs and low productivity [76].

## 4. Materials and Methods

### 4.1. Sample Preparation

The following chemicals were used: tetraethoxysilane (TEOS), (99%, for analysis, Fluka Chemie, Buchs, Switzerland); hexadecyltrimethyl ammonium bromide (CTAB, Sigma-Aldrich, Darmstadt, Germany); ethanol (Chimopar, Bucharest, Romania); 2-methoxyethanol (99%, for analysis, Sigma-Aldrich); ammonia solution 25% (Adra Chim SRL, Bucharest, Romania).

Ordered mesoporous silica materials were prepared by sol-gel synthesis in alkaline conditions, using TEOS as silica precursor and CTAB as pore forming agent, adopting traditional methods [22,77] for using solvent mixtures. As solvent, ethanol and 2-methoxyethanol were used in different proportions. A quantity of 1 g CTAB was added to 192.49 mL of distilled H_2_O under stirring. After the solution turned clear, 68 mL of alcohol was added and then 23.2 mL of aqueous ammonia solution (25%) was added, and the samples were stirred for 30 min. Ethanol was used for the first sample from each series and it was progressively substituted with 2-methoxyethanol for the next three samples in each series. After that, 4 mL of TEOS was poured into the solution slowly under stirring. Stirring continued for 3 h. After about 20 h, the solid product was recovered by filtration and washed several times with distilled water with repeated filtrations until the pH of the washing water approached the pH value of the distilled water. The samples were dried at room temperature until the next day, then further dried for several hours at 90 °C. CTAB was removed by calcination at 550 °C during 6 h, after a heating ramp with 1 °C/min. The sample names and synthesis parameters are shown in Table 4.

### 4.2. Characterization Methods

FTIR spectra were taken on KBr pellets with a JASCO–FT/IR-4200 apparatus. Samples after drying (the xerogel samples) as well as after calcination were studied. N_2_ physisorption measurements were done at 77 K using a Quantachrome Nova 1200e apparatus. Prior to the analysis the samples were dried and degassed in vacuum at 80 °C for 4 h.

The surface area was determined by the BET (Brunauer–Emmet–Teller) equation in the relative pressure range (P/P_0_) 0.01–0.25. Pore size distribution was evaluated with BJH (Barrett-Joyner-Halenda) and DFT (Density Functional Theory) methods. Total pore volumes were determined using the relative pressure point closest to 1.

Thermal measurements were performed on a Setaram LabsysEvo (Lyon, France) TG-DSC system, in flowing (90 mL/min) high purity (99.999%) synthetic air (20% O_2_ in N_2_) atmosphere. Samples were weighed into 100 μL Al_2_O_3_ crucibles (the reference cell was empty) and were heated from 25 °C to 800 °C with a heating rate of 10 °C/min. The obtained data was blank corrected and further processed with the software Calisto Processing, ver. 2.08. The temperature scale and calorimetric sensitivity were calibrated by a multipoint calibration method in which seven different certified reference materials were used to cover the thermal analyzer’s entire operating temperature range.

Transmission electron microscopy (TEM) investigation was carried out on a Philips CM20 transmission electron microscope equipped with LaB_6_ electron gun operating at 200 kV. The samples for TEM were prepared by drop-drying suspensions on holey carbon foil coated copper grids.

Small-angle neutron scattering measurements were performed on the YuMO time-of-flight instrument operating at the IBR-2 pulsed reactor source in Dubna, Russia [78,79]. The scattered neutrons were detected using the time-of-flight method by a two-detector set-up with ring wire detectors [80]. Measurements were performed on dry powders at room temperature. Corrections for transmission, background and empty aluminium container scattering were performed using SAS software [81].

Ultrasmall-angle neutron scattering measurements were performed on a double-bent crystal instrument MAUD operating at the thermal channel of the LVR15 10 MW research reactor in Řež, Czech Republic [82].

### 4.3. Metal Ion Adsorption Measurements

For the metallic ion adsorption experiments, 1000 mg/L metallic ion solution in HNO_3_ 0.5 mol/L (Merck standard solution) was used. All other metallic ion solutions were prepared from this solution with appropriate dilution. The pH of the sample solutions was pH 5 and was adjusted by using NaOH buffer solutions with the concentration in the 0.05–0.2 M range, measured using a Mettler Toledo Seven Compact S210 pH meter. Distilled water was used in all experiments.

Equilibrium isotherms were constructed using 0.1 g adsorbent materials placed in 25 mL Cu(II) solution at different initial concentrations (5–250 mg/L), and 25 mL Pb(II) solution at different initial concentrations (5–500 mg/L), at room temperature (25 ± 1 °C) and with a stirring time 2 h, using a thermostated shaker bath Julabo SW23, and shaking speed 200 rpm. The equilibrium time for isotherms adsorption was determined varying the time in the range 15–180 min for a Me(II) ions concentration of 10mg/L. After determination of the residual concentration of the metallic ions and the adsorption capacity, it was found that after 2 h the adsorption capacity has a constant value. After stirring, the samples were separated trough filtration. The residual concentration of the metallic ions in filtrate was analysed by atomic absorption spectrometry using a Varian SpectrAA 280 Fast Sequential Atomic Absorption Spectrometer with an air-acetylene flame.

## Figures and Tables

**Table 1:** Textural parameters of the calcined MCM-41 samples.

Sample	BET Surface Area (m²/g)	BET Constant	Pore Width (DFT) (nm)	Mean Pore Size (BJH) (nm)	Mean Pore Size (BJH) (nm)	Total Pore Volume (cm^3^/g)	Df (Adsorpt.)	Df (Desorpt.)
MeOE-0%-RT	1793	10	3.53	3.30	3.07	0.961	2.846	2.876
MeOE-25%-RT	1540	12	3.53	3.39	3.06	0.933	2.762	2.830
MeOE-50%-RT	1620	13	3.53	3.41	3.34	1.013	2.761	2.828
MeOE-75%-RT	1547	15	3.53	3.41	3.31	0.981	2.764	2.839
MeOE-0%-50C	1568	13	3.53	3.39	3.07	0.998	2.750	2.864
MeOE-25%-50C	1446	15	3.53	3.40	3.04	0.946	2.757	2.865
MeOE-50%-50C	1428	12	3.53	3.06	3.06	1.016	2.750	2.864
MeOE-75%-50C	1074	24	3.78	3.39	3.31	0.804	2.738	2.822

**Table 2:** Langmuir, Freundlich and Sips isotherm parameters for Cu(II) ions adsorption on sample MeOE-75%-RT and Pb(II) on MeOE-0%-RT.

Adsorbent /Metal Ion	*q*_m,exp_ (mg/g)	Langmuir Isotherm			Freundlich Isotherm			Sips Isotherm			
		*q_L_* (mg/g)	*K_L_*	R^2^	*K_F_* (mg/g)	1/*n*_F_	R^2^	*K_S_*	*q_S_* (mg/g)	1/*n*_s_	R^2^
MeOE-75%-RT /Cu^2+^	9.7	7.3	0.022	0.988	1.14	0.41	0.978	0.0026	10.2	0.3	0.990
MeOE-0%-RT /Pb^2+^	18.8	26.3	0.065	0.993	0.7	0.5	0.963	0.0061	22.2	0.6	0.995

**Table 3:** Comparison of the Me(II) adsorption capacity of the prepared mesoporous silica samples to other adsorbent materials.

Sorbent	Metal Ion	Adsorption Capacity (mg/g)	Reference
Kaolin	**Pb(II)**	4.5	[50]
activated carbon		6.7	[50]
magnetic chlorapatite nanoparticles		238	[51]
zeolite		9.9	[52]
thiol functionalized iron-oxide loaded FDU-12 mesoporous silica		287	[53]
Fe_3_O_4_@carboxymethyl-cellulose		152	[54]
Fe_3_O_4_@SiO_2_@DMSA		50.5	[55]
Fe_3_O_4_@SiO_2_@TSD		417	[56]
Fe_3_O_4_@SiO_2_ -NH_2_		76.6	[57]
pretreated *Aspergillus niger*		32.6	[58]
maghemite nanoparticle		68.9	[59]
magnetite nanoparticles		37.3	[60]
TiO_2_ nanoparticles		21.7	[61]
Al_2_O_3_ nanoparticles		41.2	[61]
MgO nanoparticles		148	[61]
Chitosan/graphene oxide		461	[62]
silica@ketoenol-pyrazole		41.8	[63]
ZnCl_2_-MCM-41		479	[64]
EDTA/SBA-15		273	[65]
waste silica coated by iron oxide		8.2	[66]
silica-magnetite composite		14.9	[67]
citrate coated SPION		58.9	[68]
gelatin-siloxane hybrid		3.75	[11]
chitosane-alginate hydrogel		85	[9]
thiol functionalized silica/magnetite		0.8	[69]
magnetic nano-zeolite		476.1	[70]
nano-silica made of *Saccharum officinarum*		148	[71]
**Calcined MCM-41**		18.8	**Present paper**
zeolite	**Cu(II)**	8.5	[52]
Fe_3_O_4_@SiO_2_ -NH		29.9	[57]
pretreated *Aspergillus niger*		28.7	[58]
maghemite nanoparticle		34.0	[59]
magnetite nanoparticles		10.8	[60]
TiO_2_ nanoparticles		50.2	[61]
Al_2_O_3_ nanoparticles		47.9	[61]
MgO nanoparticles		149.1	[61]
Chitosan/graphene oxide		423.8	[62]
silica@ketoenol-pyrazole		76.9	[63]
waste silica coated by iron oxide		3.4	[66]
magnetic nano-zeolite		59.9	[70]
steel slag/CNT composite		132.8	[72]
bifunctional silica nanospheres		139.8	[73]
nanosilica/nanopolyaniline		108	[74]
nanosilica/crosslinked nanopolyaniline		105	[74]
gelatin-siloxane hybrid		1.76	[11]
core-shell magnetite-silica NP		41	[75]
**Calcined MCM-41**		9.7	**Present paper**

**Table 4:** Synopsis of the synthesized samples.

Sample Name	CTAB (g)	TEOS (mL)	Synthesis Temperature	H_2_O (mL)	Ethanol (mL)	2-Methoxyethanol (mL)
MeOET-0%-RT	1	4	r.t.	192.5	68	0
MeOET-25%-RT	1	4	r.t.	192.5	51	17
MeOET-50%-RT	1	4	r.t.	192.5	34	34
MeOET-75%-RT	1	4	r.t.	192.5	17	51
MeOET-0%-50C	1	4	50 °C	192.5	68	0
MeOET-25%-50C	1	4	50 °C	192.5	51	17
MeOET-50%-50C	1	4	50 °C	192.5	34	34
MeOET-75%-50C	1	4	50 °C	192.5	17	51

## Data Availability

Experimental data are available from the authors.
